# List of Reviewers

**DOI:** 10.2471/BLT.21.970121

**Published:** 2021-01-01

The Editorial Team would like to thank all those who gave generously of their time and expertise in reviewing the papers for the *Bulletin of the World Health Organization *in 2020. We apologize to anyone whose name has been inadvertently omitted from the following list:

## A

Abbara, Aula

Abd El-Wahab, Ekram

Abdelhai, Rehab

Abeykoon, Palitha

Abo-Rahma, Alyaa

Aboubaker, Samira

Aboushady, Ahmed

Aboussaleh, Youssef

Abramowitz, Sharon

Adams, Rosmond

Adegbosin, Adeyinka

Adewoyin, Yemi

Aduo-Adjei, Kofi

Aggarwal, Neil

Agrawal, Priyanka

Ahmad, Irshad

Ahmed,, Syed Masud

Akin, Ayse

Aklilu, Abenezer

Akram, Hammad

Al Balkhy, Hanan

Alaama, Ahmed Sabry

Alabbas, Amr

al'Absi, Mustafa

Albarqouni, Loai

Alcalde Rabanal, Jackeline

Alhomaizi, Dalal

Alkoronky, Asi

Allanson, Emma

Allen, Koya

Allen, Luke

Al-Nsour, Mohannad

Alonso, Wladimir

Aloosh, Mehdi

Amadi, Hippolite

Amato, Stas

Amiri, Masoud

Amul, Gianna Gayle

Anderson, Claire

Andreuccetti, Gabriel

Andualem, Tenaw

Ansari, Shamshul

Anyetei-Agama, Margaret

Ara, Iffat

Arab-Zozani, Morteza

Aref, Samin

Ashigbie, Paul

Asi, Yara

Asokan, G

Atique, Suleman

Auger, Nathalie

Awan, Haroon

Awan Najma

AyalaQuintanilla, Beatriz Paulina

Aye, Khin

Azuma, Kenichi

## B

Babatunde, Opeyemi

Babor, Thomas

Baek, Yeji

Baeten, Jared

Baeza, Juan

Bakshi, Ravleen

Banda, Geoffrey

Bang, Abhay

Baral, Stefan

Barkley, Shannon

Barraclough, Simon

Basnayake, Anoma

Bassat, Quique

Basu, Saurav

Baxi, Maulik

Becerra, Carlos

Becker, Gregory

Beiki, Omid

Bell, Colin

Bell, Sue Anne

Bellingham-Young, Denise

Belsare, Aniruddha

Bennett, Jo Anne

Bentley, Rebecca

Bernard, Chawo Silenou

Bhadelia, Afsan

Bhambere, Sailee

Bhandari, Dinesh

Bharati, Premananda

Bharti, Omesh

Bianchi, Renzo

Bijlmakers, Leon

Billah, Baki

Block, Susan

Boakye, Alex

Boci, Arian

Bolgiano, Barbara

Bolton, Kirsty

Bonfoh, Bassirou

Bonnin Roca, Jaime

Borame Dickens, Sue

Borghi, Elaine

Boyce, Ross

Braitstein, Paula

Brasola, Davide

Brennan, Siobhan

Brickley, Elizabeth

Bright, Tess

Bruno, Alfredo

Bucagu, Maurice

Bucher, Bernard

Bui, Linh

Burmen, Barbara

Butchart, Alexander

Byskov, Jens

## C

Caballes, Alvin

Cakmak, Fatih

Cameron, Kathleen

Canals, Maria Luisa

Caraher, Martin

Casbon, James

Caskey, Fergus

Castrejon-Perez, Roberto Carlos

Castro, Rodolfo

Chadha, Shelly

Chakraborty, Rhyddhi

Chami, Goylette

Chanchitprich, Chaunjit

Chandra Mathur, Shiv

Chang, Chee Tao

Chang, LA3pez Roberto

Changula, Katendi

Charles, Lauren

Charlesworth, Karen

Charo, Alta

Chaudhry, Rabia

Chaw, Liling

Chen, Bin

Chen, Mark

Chen, Solomon Chih-Cheng

Chen, Yingyao

Chico-Sáchez, Pablo

Chompook, Pornthip

Chow, Eric

Chowell, Gerardo

Cieza, Alarcos

Citron, Isabelle

Coates, Harvey

Cochrane, Logan

Codyre, Patricia

Cohn, Jennifer

Comendant, Rodica

Cometto, Giorgio

Connor, Jennie

Çöplü, Nilay

Cornejo, Javiera

CA'tA(c), Anne-Marie

Coviello, Enzo

Crainey, James

## D

Dada, Sara

Daher, Michel

Dalby, Simon

Darney, Blair

Dartnall, Elizabeth

Daugherty, Sarah

Deboutte, Danielle

Deckert, Andreas

DeFelice, Nicholas

De Maeseneer, Jan

Deribe, Kebede

Devine, Angela

Dewey, Deborah

Dey, Sukhen

DomA-nguez, Angela

Dos Santos, Vagner

Downs, Jennifer

Drumright, Lydia

## E

Ebiowei Orubu, Sam

Ehsan, Nermine

Elafros, Melissa

Eliakimu, Eliudi

El-Omar, Emad

Engelhardt, Katrin

Erkhembayar, Ryenchindorj

Escalante, Socorro

Espinosa Gonzalez, Ana Belen

Ezziane, Zoheir

## F

Fabreau, Gabriel

Faithpraise, Fina

Farrington, Paddy

Fasehun, Luther-King

Fei, Gaoqiang

Fellone, Lucio

Feng, Xing Lin

Ferdinando, Ruwan

Fernández, Emiliano

Fernando, Gabriela

Ferrari, Stefano

Ferretti, Ceres

Figueiredo, Alexandre

Fitzgerald, Gerry

Ford, Nathan

Forde, Andrea

Fouchier, Ron

Franses, Philip Hans

Fraser, Joy

Freedman, David

Friedman, Joseph

Frisch, Miriam

Frongillo, Edward

Fu, Peipei

Fullybright, Rudolf

Furin, Jennifer

## G

Gable, Lance

Gadde, Praveen

Gan, Heng

Gay, Noellie

Ge, Yang

Gemmill, Alison

George, Asha

George, Charles

Ghosh, Pramit

Gibson, Roger

Gigante, Valeria

Gios, Lorenzo

Glaziou, Philippe

Gleason, Kelsey

Glushkova, Natalya

Glynn, Judith

Goel, Varun

Gokani, Nikhil

Gonzalez, JeanPaul

González, Edgar

Goodchild, Mark

Goos, Cornelius

Gopal, Nikhil

Gorina, Yelena

Goryakin, Yevgeniy

Goudarzian, Amir Hossein

Govil, Dipti

Goyanka, Roopali

Gozzer, Ernesto

Gqaleni, Nceba

Greening, Bradford

Greenwood-Basken, Maureen

Griffiths, Elwyn

Gryseels, Charlotte

Guedes, Diego

## H

HabibiSaravi, Reza

Haddad, Samira

Haileamlak, Abraham

Halstead, Scott

Hammad, Faris

Hansen, Nina

Hanson, Claudia

Hanssen, Odd

Haq, Muhammad Ijaz-Ul

Hariga, Fabienne

Harris, Joseph

Harris-Roxas, Ben

Hasanpoor, Edris

Hatefi, Arian

Hayford, Kyla

He, Daihai

He, Zonglin

Hedt-Gauthier, Bethany

Heller, David

Hensel, Lukas

Hipple Walters, Bethany

Hirschhorn, Lisa

Hlongwane, Tsakane

Hlope, Londiwe

Hogerzeil, Hans

Hollingworth, Samantha

Holm, Michelle

Holmes, Wendy

Honorio, Nildimar

Horne, Simon

Hsieh, Chee-Ruey

Hughes, Alex

Huish, Robert

Humayun, Ayesha

Hussain, Rabia

Hutin, Yvan

## I

Isabirye, Nathan

Ishizuka, Aya

Isoda, Norikazu

Iverson, Katherine

## J

Jahangeer, Syed Muhammad Ashraf

Jain, Vageesh

Jama, Mohamad

Jani, Anant

Jarhyan, Prashant

Jarju, Sheikh

Javid, Babak

Jayanna, Krishnamurthy

Jennings, Bruce

Jia, Haimei

Johannessen, Asgeir

John, Rijo

Johnson, Leigh

Jones, Alexandra

Jones, Sam

Jonnagaddala, Jitendra

Joos, Olga

Joss, Meltem

## K

Kaewanuchit, Chonticha

Kalanda, Boniface

Kale, Harshavardhan

Kalita, Anuska

Kanamori, Shogo

Kane, Jeremy

Kangbai, Jia

Karalasingam, Shamala

Kasper, Toby

Kaur, Sharon

Kayiwa, Joshua

Keni, Bhalachandra

Khachadourian, Vahe

Khachfe, Hussein

Khalifa, Mohamed

Khan, Rabia

Khan, Selim

Khanna, Kajal

Kholoud, Kahime

Khorrami, Zahra

Khosla, Rajat

Khosrawipour, Tanja

Kickbusch, Ilona

Kim, Jin-hwan

Kinder, Karen

King, Jessica

Klausner, Jeffrey

Ko, Hansoo

Kovach, Kevin

Krasnik, Allan

Krentel, Alison

Kriebel, David

Krishnamurthy, Ramesha

Kuivasniemi, Outi

Kulkarni, Shrikant

Kumar, Ramya

Kutalek, Ruth

Kwamie, Aku

Kwan Yu, Heng

## L

Laaser, Ulrich

Laborde, Gladys Mae

Lachaud, James

Lahariya, Chandrakant

Land, Mary-Anne

Langlois, Etienne

Lansingh, Van

Lapitan, Jostacio

Larg, Allison

Laroche, Lorie

Lartey, Stella

Lawson, David

Lee, Brian

Lee, David

Leeder, Stephen

Leinster, Sam

Leslie, Hannah

Leufkens, Hubert

Leyva-Flores, RenA(c)

L'Huillier, Arnaud

Li, Jih-Heng

Li, Qingfeng

Li, Wenzhen

Liberman, Jonathan

Lim, Ka keat

Lima, JosA(c)

Lin, Vivian

Liu, Dianchang

Liu, Jufen

Liu, Xiaoyun

Liu, Ying

Long, Kanya

Lotfizadeh, Masoud

Lovric, Zvjezdana

Luby, Stephen

Lugo Robles, Roberta

Lumagbas, Lily Beth

Lumpkin, Murray

Lunt, Neil

Luong, Chinh

Luthra, Megha

Luyendijk, Rolf

Luz, Alia Cynthia

## M

M'buyamba, Jean Rene

Macinko, James

MacIntyre, Raina

Maffioli, Elisa

Magnusson, Roger

Mahdian, Mehrdad

Malqvist, Mats

Malta, Monica

Mandal, Shyamapada

Mansilla, Cristián

Mariotti, Silvio

Maroa, Jenniffer

Marsh, Vicki

Marston, Hannah

Marten, Robert

Martin Herz, Susanne

Martin-Ortega, Olga

Marx, Michael

Maswime, Salome

Matanje Mwagomba, Beatrice

Mathonnat, Jacky

Mattison, Donald

May, Margaret

Mbabazi, Pamela

McBride, David

McDonald, Matt

McGrady, Benn

McKenna, Anne

Meinck, Franziska

Mejia, Nelly

Mendes Jorge, Margarida

MA(c)ndez, Claudio

Mendis, Shanti

Metelmann, Isabella

Methven O'Brien, Claire

Mhazo, Tichatyei

Michie, Susan

Miller, F. DeWolfe

Miller, Tina

Milnor, Jacob

Miranda, Manuel Albela

Mirza, Muzna

Mohammad, Yousser

Mohammed, Mahmood

Mohammed, Menbeu

Moitinho de Almeida, Maria

Mokhtar, Amany

Montserrat, Emili

Moroz, Paul

Morris, Michael

Morse, Tracy

Mounien, Lourdes

Mounsey, Sarah

Mpyet, Caleb

Mujica, Oscar

Muraduzzaman, A K M

Murakami, Michio

Murphy, Richard

## N

Nair, Pradeep

Nambiar, Devaki

Nasiri, Saeideh

Neethling, Ian

Nemser, Bennett

Nena, Evangelia

Ng, Chiu-Wan

Nicol, Dianne

Nilsson, Peter

Nkangu, Miriam

Nkya, Siana

Noman, Hanan

Nongkynrih, Baridalyne

Novotny, Thomas

Nunes, João

## O

Odezugo, Gertrude

Odjidja, Emmanuel

Ogedegbe, Olugbenga

Okeke, Iruka

Okereafor, Kenneth

Oldenburg, Catherine

Oliver, Emily

Oppong, Joseph

Ortiz-Prado, Esteban

Otanez, Marty

Ozanne-Smith, Joan

Öztaş, Tülin

## P

Palenfo, Dramane

Pang, Jun

Pannenborg, Ok

Paredes-SolA-s, Sergio

Parvez, Mohammad

Patcharanarumol, Walaiporn

Patel, Pragna

Patil, Rutuja

Pattinson, Robert

Peeling, Rosanna

Peeters, Koen

Penm, Jonathan

Penney, Tarra

Peterman, Thomas

Peterson, Chris

Petrea, Ionela

Pham, Bang

Phillips, J. Craig

Piffaretti, Jean-Claude

Pineau, Pascal

Pinto Junior, Vitor

Pitukthanin, Anuk

Polis, Chelsea

Pont, Joerg

Popay, Jennie

Potharaju, Nagabhushana

Prasertchai, Araya

Pratiti, Rebecca

Pullar, Jessie

Putri, Septiara

## Q

Quigley, Paula

Quintana, MarA-a Gabriela

## R

Rahman, Md Mizanur

Rahman, Motiur

Rai, Anjana

Rai, Saraswathi Bina

Ramanan, Mahesh

Randrianasolo, Laurence

Ranga, Aniruddh

Ranjan, Alok

Ranzani, Otavio

Rasheduzzaman, Md

Ratnayake, Ravi

Ravinetto, Raffaella

Reacher, Mark

Recidoro, Zenaida

Reddy, Ravi

Renouf, Tia

Resnikoff, Serge

Riboulet-Zemouli, Kenzi

Ricarte, Ivan

Ricca, Jim

Riccardo, Flavia

Rittmeyer, Miriam

Riva, Nicoletta

Rodewald, Lance

Rodriguez, Francisca

Rodriguez Almagro, Julian

Rodriguez Llanes, Jose Manuel

Rodriguez Manzano, Jesus

Rogier, Eric

Rokicki, Slawa

Roll-Hansen, Dag

Rollins, Nigel

Rosario, Barbara

Rosinger, Asher

Rouhani, Shada

Roy, Subarna

Rudd, Kristina

Rudge, James

Ruiz, Fabrice

Rupprecht, Charles

## S

Sahastrabuddhe, Sushant

Saif-Ur-Rahman, K M

Saito, Tomoya

Sandor, Janos

Sarla, Gurmeet

Saroj, Rakesh 

Sayed, Shaheen

Schaaf, Marta

Schuwirth, Lambert

Seeley, Janet

Seff, Ilana

Selleck, Paul

Serruya, Suzanne

Setshegetso, Naomi

Shafiee, Elham

Shah, Shahid

Shakur, Yaseer

Shan, Jing

Sharifi, Hamid

Sharma, Vidushi

Shenoy, Niraj

Shobugawa, Yugo

Shroff, Zubin

Siddiqui, Saiful

Silver, Lynn

Simeon, Donald

Singh, Sandeep

Singhal, Sonica

Sitas, Freddy

Sithey, Gyambo

Skrip, Laura

Sogarwal, Ruchi

Sohn, Yeowon

Sommer, Marni

Song, Liting

Spiegel, Paul

Sripada, Kam

Stambler, Ilia

Stelma, Foekje

Stöver, Heino

Su, Huanxing

Sudandiradas, Robin

Sule, Waidi

Sultana, Papia

Sun, Mei

Sunyoto, Temmy

Susanna, Dewi

Svedberg, Peter

## T

Taderera, Bernard

Talkington, Kathy

Tam, Clarence

Tanaka, Junko

Tandale, Babasaheb

Tanno, Luciana

Tawhidul, Sheikh

Taylor, Elizabeth

Taylor, Sebastian

ten Have, Henk

Terrones-Lozano, Alejandro

Teruya Uchimura, Liza

Thapa, Rajshree

Thein, Swee Lay

Theis, Torsten

Tholandi, Maya

Thoma, Marie

Thomas-Bosco, Rebekah

Thompson, Jacqueline

Thompson, Michael

Thow, Anne Marie

Tintinalli, Judith

Togami, Eri

Torgerson, Paul

Torres-Rueda, Sergio

Treleaven, Emily

Tristao, Rosana

Trost, Matthias

Truche, Paul

Tsiachristas, Apostolos

Tsutsumi, Atsuro

Tuyet, Nguyen

## U

Ujang, Uznir

Ukhayakumar, Venkatachalam

## V

Van Remoortel, Hans

van Weel, Chris

Vanoni, Matia

Veillard, Jerremy

Venter, Francois

Vento, Sandro

Verma, Akalesh

Vermeiden, Tienke

Vigliecca, Nora

Villalobos, Aremis

Villanueva, Antonio Fredelindo

Vitek, Megan

von Gaudecker, Jane

## W

Walley, John

Wamuti, Beatrice

Wang, Michael

Wang, Weibing

Wang, Xin

Wangdi, Kinley

Ward, Catherine

Ward, John

Watts, Nick

Weaver, Marcia

Webster, Elizabeth

Weerasuriya, Krisantha

Weiss, Helen

Werner, Kalin

White, Franklin

Wi, Teodora

Winget-Gibson, Jean

Winter, Sam

Wirtz, Veronica

Witt, Clara

Wong, Eliza Lai-yi

Woo, Jean

Woods, Catherine

Wozniak, Teresa

## X

Xin, Tianyi

Xu, Ke

Yakob, Laith

Yakutcan, Usame

Yombi, Jean Cyr

## Z

Zeitlin, Jennifer

Zeng, Wu

Zerbo, Alexandre

Zermoglio, Fernanda

Zhang, Yan-Bo

Zhou, Chengchao

Zhou, Fangjun

Zhou, Wei

Zimbudzi, Edward

Zimmerman, Mark

Zin, Thant

Zolnikov, Tara

Zou, Guanyang

Zulu, Tryphine

